# Antioxidant activity and total phenolic compounds of Dezful sesame cake extracts obtained by classical and ultrasound-assisted extraction methods

**DOI:** 10.1002/fsn3.118

**Published:** 2014-05-18

**Authors:** Reza Esmaeilzadeh Kenari, Fatereh Mohsenzadeh, Zeinab Raftani Amiri

**Affiliations:** Department of Food Science and Technology, Sari Agriculture and Natural Resources UniversitySari, Mazandaran, Iran

**Keywords:** DPPH, Folin–Ciocalteu, FRAP, maceration, sesame cake, sonication, *β*-carotene bleaching

## Abstract

Sesame cake is a by-product of sesame oil industry. In this study, the effect of extraction methods (maceration and sonication) and solvents (ethanol, methanol, ethanol/water (50:50), methanol/water (50:50), and water) on the antioxidant properties of sesame cake extracts are evaluated to determine the most suitable extraction method for optimal use of this product. Total phenolic content is measured according to the Folin–Ciocalteu method and antioxidant activities of each extract are evaluated with the 2,2-diphenyl-1-picrylhydrazyl (DPPH), *β*-carotene bleaching, and ferric reducing/antioxidant power (FRAP) methods. The highest amount of total phenolic compounds is observed in ethanol-ultrasonic extract with the amount of 88.89 mg/g gallic acid equivalent. Methanol-ultrasonic extract with the amount of 88.475% indicates the highest activity in scavenging DPPH free radicals. In *β*-carotene-linoleic acid system, ethanol-ultrasonic extract indicates the highest inhibition percent of 45.64. In FRAP assay, ethanol/water (50:50)-maceration and ethanol/water (50:50)-ultrasonic extracts with the absorption of 1.132 and 1.0745 nm indicate the highest antioxidant activity.

## Introduction

Oxidation is a chemical reaction that produces free radicals. These radicals initiate a chain reaction that may be lead to cell damage or death (Sies 1997). Free radicals react with lipid and cause lipid peroxidation in food that affected its color, odor, texture, and quality (Ferreira et al. 2007). In addition, the toxic substances produced lead to mutagenic and carcinogenic effects (Yagi 1990). Antioxidants terminated these reactions with elimination of free radicals and prevent other oxidative reactions by oxidizing themselves (Sies 1997). So antioxidants are added to delay food oxidation (Ito et al. 1982). Antioxidants are divided into synthetic and natural groups. Due to the undesirable effect of synthetic antioxidants, the replacement of a natural one from plant sources was investigated (Hwang et al. 2001). Sesame (*Sesamum indicum* L.) is from *Pedaliaceae* family, *Sesamum* genus, and *S. indicum* species. Chemical compounds of sesame indicated that sesame seed is a rich source of oil (44–58%), protein (18–25%), carbohydrate (13.5%), and ash (5%) (Kamal-Eldin and Appelqvist 1994; Yoshida 1994; Mohamed and Awatif 1998; Shyu and Hwang 2002; Kahyaoglu and Kaya 2006). Budowski (1964) reported that sesame oil is more stable to oxidation in comparison with others. Wide studies were done about the health effects of sesame (Kang et al. 1999). They show hypocholesterolemic, anticancer, and antiaging effects (Sugano et al. 1990; Yamashita et al. 1995; Kang et al. 1999). Lignans and lignan glycosides of seed, oil, and cake are responsible for the above properties (Suja et al. 2004). Lignan glycosides that mainly existed in the sesame-defatted cake are polar antioxidants. The most important sesame lignan glycosides are sesaminol glucosides, pinoresinol glucosides, and sesamolinol glucosides (Kuriyama et al. 1993). Sesame cake is a by-product of sesame oil industry. In some countries, sesame cake is discarded or used as poultry feed. Primary studies show that a significant amount of antioxidant compounds still existed in the sesame cake (Mohdaly et al. 2011). There are some reports about antioxidant activity of sesame seed and cake, but there is no report about the effect of extraction methods on extraction of antioxidant compounds, therefore optimizing extraction process to reach to the maximum amount of these compounds is the main aim of this study. Choice of a method to extract the active ingredients with maximum efficiency and the highest purity depends on the nature of the ingredients, thermal stability, and the nature of the processed raw materials (Shirsath et al. 2012). Solvent extraction is often used to separate the antioxidant's extraction efficiency, which is dependent on the solvent and extraction method due to the differences in polarity of the antioxidants. Several extraction techniques have been reported for the extraction of phenolic compounds from different matrices using solvents with different polarities (Goli et al. 2005). Recently new extraction techniques such as ultrasound-assisted extraction, microwave-assisted extraction, and supercritical fluid extraction have been reported (Wang and Weller 2006). Among these methods, ultrasound-assisted extraction is one of the cheapest, simplest, and the most effective one which can increase the efficiency of ingredients’ extraction, reduce extraction time, and provide more processing efficiency (Chen et al. 2010). Sound waves with the frequency higher than 20 kHz cause mechanical vibrations in solids, liquids, and gases (Jakopic et al. 2007). Cavitation phenomenon as a result of ultrasonication defined as generated, growth, and collapse of the gas steam-filled bubbles in a liquid. Cavitation causes rotational flow and disturbance in a liquid that lead to significant increase in mass transfer (Goli et al. 2005). So in this study, antioxidant properties of sesame cake extract obtained by maceration and ultrasonic-assisted extraction methods using several solvents such as methanol 100%, ethanol 100%, ethanol/water (50:50), methanol/water (50:50), and water 100% will be compared to optimize extraction method.

## Materials and Methods

### Materials

Sesame cake was purchased from a sesame oil production factory located in Shiraz, Iran. Folin reagent prepared from Merck (Darmstad, Germany) was obtained. DPPH (2,2-diphenyl-1-picrylhydrazyl) prepared from Sigma-Aldrich (St. Louis, MO) was purchased, and in the ferric reducing/antioxidant power (FRAP) method, sodium phosphate buffer (pH 6.6), potassium ferricyanide, ferric chloride (Sigma), and trichloroacetic acid (Merck) were used. In *β*-carotene bleaching method, *β*-caroten, linoleic acid, and Tween 40 (Sigma) were used. Butylated hydroxyanisole (BHA) was used as standard antioxidant and it was prepared from TITRAN.

### Maceration extraction

The different solvents (ethanol, methanol, water, ethanol/water [50:50], methanol/water [50:50]) were added to powdered sesame cake in the ratio of 10:1 (Chang et al. 2002) and the resulting mixtures were shaken overnight to extract sesame cake′s phenolic compounds. After 24 h, the extracts were filtered through Whatman No. 42 filter paper to separate sesame cake particles. The solvents were completely evaporated in an oven at 40°C. Finally, they were placed in a refrigerator.

### Ultrasound-assisted extraction

The ultrasound-assisted extraction procedure was used for the extraction of sesame cake with the same solvents and at the same ratio. The mixture was sonicated for 45 min (Goli et al. 2005) in an ultrasonic bath (Elma s 30 H model, total Power Consumption: 280W, Heating Power: 200W, operating at 37 kHz frequency and internal dimensions: 198 × 106 × 50 cm). The temperature was controlled and maintained at 30°C by circulating water. The extracts were filtered and the remaining steps were similar to those of the previous method.

### Total phenolic content (TPC)

The amounts of phenolic compounds in the extracts were determined according to the Folin–Ciocalteu method (Savitree et al. 2004; Pourmorad et al. 2006) and results were expressed as mg/g gallic acid equivalents (GAE). Concentration of 1 mg/mL of each sesame cake, extracts were prepared in their own solvents and 0.5 mL of each sample mixed with 2.5 mL of a 10-fold diluted Folin–Ciocalteu reagent and 2 mL of 7.5% sodium carbonate solution. Then the samples were kept for 30 min at room temperature and at the end the absorbance was read spectrometrically (T80 + UV/VIS spectrophotometer) at 760 nm.

### DPPH˙ radical-scavenging activity

The capacity to scavenge DPPH free radicals was measured according to the method of Hatano et al. (1988). A quantity of 0.3 mL of each extract with a different concentration was mixed with 2.7 mL of DPPH radicals (6 × 10^−5^ mol/L) solution. The mixture was shaken vigorously and maintained in a dark place for 60 min, until the absorbance values reach the steady state. The reduction in DPPH radical was measured by monitoring the decrease in absorption at 517 nm. DPPH scavenging effect was calculated as a percentage of DPPH discoloration using the equation:


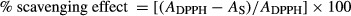


where *A*_S_ is the absorbance of the solution when the extracts have been added at different concentrations and *A*_DPPH_ is the absorbance of the DPPH solution. The extract concentration providing 50% inhibition (EC_50_) was calculated from the graph of scavenging effect percentage against extract concentration in the solution. BHA methanolic solutions were used as standards.

### *β*-Carotene bleaching system

The antioxidant activity of sesame cake extracts was also determined by measuring the inhibition of hydroperoxides formed from linoleic acid oxidation (Dapkevicius et al. 1998). *β*-carotene/linoleic acid solution was prepared according to the following method: 0.5 mg *β*-carotene was dissolved in 1 mL chloroform (high-performance liquid chromatography grade), then, 25 *μ*L linoleic acid and 200 mg Tween40 were added. Chloroform was totally evaporated using rotary vacuum evaporator. Then, 100 mL of distilled water saturated with oxygen was added and the contents shaken vigorously, 2.5 mL of the above solution was transferred to the test tube and 350 *μ*L of each extract (with a concentration of 2 g/L dissolved in their own solvent) was added. All of the above procedures were done for blanks (*β*-carotene stock solution in addition to the solvents). All samples were put into a water bath for 120 min at 50°C. The absorbance values of samples were read spectrophotometrically at 470 nm and were taken immediately at zero time and after 120 min. Antioxidative capacities of the extracts were expressed as percentage inhibition (Duarte-Almeida et al. 2006).


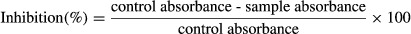


### Ferric reducing/antioxidant power

The FRAP was evaluated according to the method of Oyaizu (1986). A quantity of 2.5 mL of the extract solution was combined with 2.5 mL of 200 mmol/L sodium phosphate buffer (pH 6.6) and 2.5 mL of 1% potassium ferricyanide. The above mixture was incubated at 50°C for 20 min. After that, 2.5 mL of 10% trichloroacetic acid (w/v) was added, then, the mixture was centrifuged at 116.272 *g* for 8 min (HERMEL Z × 200A centrifuge). Five milliliters of the upper layer was mixed with 5 mL of deionized water and 1 mL of 0.1% of ferric chloride. Finally, the absorbance values of the solutions were read spectrophotometrically at 700 nm. The solution with the higher absorbance value shows higher reducing power. In this method, EC_50_ refers to the concentration of extract in the solution which shows the absorbance of 0.5. It was calculated from the graph of absorbance at 700 nm against extract concentration in the solution. To compare extract reducing power, methanolic solution of BHA was used as the standard.

### Statistical analyses

All experiments were carried out in triplicate. The analytical data were shown as mean ± standard deviation of measurements. Then, the results were subjected to one-way analysis of variance (ANOVA), the significance of mean differences was determined by Tukey's test with *α* = 0.05 using Minitab version 16.0. Excel software was used to plot the graphs.

## Result and Discussion

### Analysis of sesame cake

The chemical compositions of sesame cake were analyzed and are shown in Table 1.

**Table 1:** Chemical composition of sesame cake

Moisture (%)	7.92
Fat (%)	27.83
Protein (%)	30.56
Fiber (%)	6.22
Ash (%)	5.27
Carbohydrate (%)	28.14

### Total phenolic content

As can be seen in Table 2, ethanol-ultrasound (EU) extract was the best extract, followed by methanol-ultrasound (MU), methanol-maceration (MM), and ethanol-maceration (EM). After these, ethanol/water (50:50)-ultrasound (E_50_U), methanol/water (50:50)-ultrasound (M_50_U), methanol/water (50:50)-maceration (M_50_M), and ethanol/ water (50:50)-maceration (E_50_M) extracts were at the next degree. Aqueous extracts were at the lowest degree. Ultrasound can have a positive effect on ethanol, ethanol/water (50:50), and water solvents, so that the extracts obtained from ultrasound-assisted extraction method with the above solvents can make a significant difference in terms of phenolic compounds with similar extracts obtained from the maceration method, while there were no significant differences among MU and MM and also between M_50_U and M_50_M. This is due to the effect of solvent properties such as vapor pressure, viscosities, and surface tension on incidence of cavitation. The value of these properties for ethanol, methanol, and water has been shown in Table 3. The effect of vapor pressure in ultrasonication is related to the production of cavitational bubbles. It means that solvents with lower vapor pressure produce fewer cavitational bubbles that require higher force to collapse. So, during extraction, plant tissues are disrupted with more intensity. On the other hand, the solvents with high vapor pressure produce more bubbles but with less force to collapse. So, these kinds of solvents are not very effective for extraction. In the case of viscosity, liquids with low viscosity are more effective, because ultrasonic intensity applied could more easily overcome molecular force of the liquids with low viscosity. In addition, a liquid with low viscosity can easily penetrate into plant texture, because of its low density and high diffusivity. Liquids’ surface tension is another feature that contributes to the formation of cavitational bubbles. In the liquids with lower surface tension, cavitational bubbles are created more easily, because ultrasonic intensity applied could more easily exceed the surface tension force (Mason et al. 1996). As can be seen in Table 3, ethanol and methanol have a similar surface tension, so we discuss about two other factors. Although viscosity of ethanol is higher than that of methanol, but because of its lower vapor pressure, bubbles require higher force to collapse, so more energy is released to disrupt plant tissue. Low viscosity of methanol leads to formation of higher cavitational bubbles but, due to their high vapor pressure, they decompose with less intensity (Hemwimol et al. 2006). In the presence of water, extraction of phenolic compounds decreased due to the high polarity of mixture, but in the presence of 50% water, extraction of phenolic compounds increased due to the relative increase in the polarity and also due to the increase in the swelling of plant tissue. In addition, the presence of water leads to a decrease in viscosity of mixture, therefore mass transfer was improved. When ultrasonification was applied, extraction of phenolic content increased in the presence of 50% water as with maceration method. In the presence of water, the intensity of ultrasonic cavitation increased due to the decrease in vapor pressure and viscosity of mixture (Rostagno et al. 2003). TPC in the aqueous extract was lower than its amount (65.02 mg/g) in the same extract of Peschel et al. (2007), but regarding methanolic extract of this study, TPC value was greater than its amount (1709 *μ*g/g) in the same extract used by Suja et al. (2005). TPC values of evening primrose cake in hydro-alcoholic (659.51 mg/g) and aqueous extract (229.63 mg/g) (Peschel et al. 2007) and *Adhatoda vasica* in hydro-alcoholic (81.51 mg/g) and aqueous extract (92.04 mg/g) (Maurya and Singh 2010) were greater than those in sesame cake hydro-alcoholic and aqueous extract in this research.

**Table 2:** Total phenolic content of sesame cake extracts

Samples	Concentration (*μ*g/mL)	Mean ± standard deviation
EM	1000	60.92b ± 3.99
MM	1000	61.21667b ± 1.047
E_50_M	1000	27.98d ± 1.53
M_50_M	1000	34.04667cd ± 3.65
WM	1000	22.03d ± 7.15
EU	1000	82.89667a ± 5.62
MU	1000	62.09b ± 0.79
E_50_U	1000	48.39bc ± 5.71
M_50_U	1000	37.24cd ± 5.38
WU	1000	30.27667d ± 10.35

Different letters in the column indicate significant differences (*P* < 0.05). EM, ethanol-maceration; MM, methanol-maceration; E_50_M, ethanol:water (50:50)-maceration; M_50_M, methanol:water (50:50)-maceration; WM, water-maceration; EU, ethanol-ultrasonic; MU, methanol-ultrasonic; E_50_U, ethanol:water (50:50)-ultrasonic; M_50_U, methanol:water (50:50)-ultrasonic; WU, water-ultrasonic.

**Table 3:** The properties of solvent used in the extraction (at 25°C), (Hemwimol et al. 2006)

Solvent	Polarity	Surface tension (mN/cm)	Vapor pressure (mmHg)	Viscosity (CP)
Methanol	5.1	22.6	127.05	0.6
Ethanol	5.2	23.7	59.02	1.2
Water	9	72.8	23.8	0.89

### DPPH radical scavenging activity

**Figure 1:**
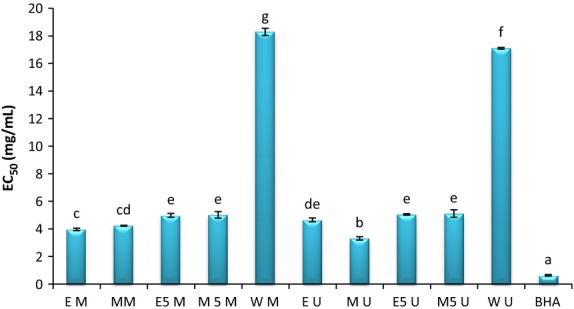
EC_50_ (mg/mL): effective concentration at which 50% of DPPH radicals are scavenged. EM, ethanol-maceration; MM, methanol-maceration; E_50_M, ethanol:water (50:50)-maceration; M_50_M, methanol:water (50:50)-maceration; WM, water-maceration; EU, ethanol-ultrasonic; MU, methanol-ultrasonic; E_50_U, ethanol:water (50:50)-ultrasonic; M_50_U, methanol:water (50:50)-ultrasonic; WU, water-ultrasonic.

Antioxidant activity of plant extracts containing polyphenolic compounds is due to their capacity to donate hydrogen atoms or electrons and free electrons (Shon et al. 2003). DPPH˙ free radical assay, is based on a single electron transfer mechanism and also hydrogen atom transfer mechanism, thus providing a better method for antioxidant capacity (Pérez-Jiménez et al. 2008). DPPH is a stable free radical with purple color, which in the presence of antioxidants changes into yellow. In general, reducing capacity of DPPH˙ free radical is determined by decreasing its absorption at 517 nm (Duh 1998). With increasing concentration or degree of hydroxylation of phenolic compounds, DPPH radical scavenging activity also increased and is defined as antioxidant activity (Zhou and Yu 2004). The whole system is done in a very low concentration due to the high sensitivity of free radicals in the presence of hydrogen donors (Iqbal et al. 2006). In this study, DPPH˙ free radical assay depends on extract concentration, extraction solvent, and extraction method. As can be seen in Table 4 antioxidant activity of all extracts increased with the increase in concentration from 1 to 10 mg/mL. These results were in agreement with Xiea et al. (2012) who mentioned that extract concentration is an effective factor in enhancing antioxidant activity. The highest antioxidant activity belonged to MU extract at the concentration of 10 mg/mL without any significant differences with BHA at the concentration of 2 mg/mL up to 10 mg/mL, but it shows significant differences with other extracts. This was in agreement with Pinelo et al. (2004) who mentioned that methanolic extract is the best extract in order to scavenge DPPH free radical. They found that although ethanol was more effective in extraction of phenolic compound, but regarding the antioxidant activity of methanolic extract, it is more than that of the ethanolic one and that of ethanolic extract is more than that of the aqueous one and could scavenge DPPH free radical. In general, DPPH free radical scavenging method is more suitable in an organic medium than in an aqueous one (Kim et al. 2002). The extract concentrations which provide 50% inhibition of free radicals are indicated with EC_50_. Therefore, the extracts with lowest EC_50_ have the largest antioxidant property. As can be seen in Figure 1, the lowest EC_50_ with 0.629 ± 0.054 mg/mL is related to BHA as standard antioxidant. MU with 3.3065 ± 0.119 mg/mL was after that. This is not in agreement with the amount of EC_50_ (150 mg/mL) in the research by Suja et al. (2005). EM, MM, and EU with 3.961 ± 0.905 mg/mL, 4.231 ± 0.03 mg/mL, and 4.654 ± 0.135 mg/mL were in the next degree. There were no significant differences in hydro-alcoholic samples. Water-ultrasonic (WU) and water-maceration (WM) with 17.100 ± 0.061 mg/mL and 18.28 ± 0.261 mg/mL had the highest EC_50_ and thus the lowest antioxidant activity was seen in the aqueous extracts. This was because of the small amount of phenolic compounds in the aqueous extract and also hydrophilic nature of extract that was in contrast with hydrophobic nature of this method.

**Table 4:** Percent Scavenging effect of sesame cake extracts

Samples Conc mg/mL	Scavenging DPPH free radicals										

	EM	MM	E_50_M	M_50_M	WM	EU	MU	E_50_U	M_50_U	WU	BHA
1	27.605 ± 0.756st	27.915 ± 0.968st	18.905 ± 0.205wxy	20.945 ± 0.841vwx	8.06 ± 0.12AB	22.675 ± 0.714uv	28.06 ± 0.127st	25.035 ± 0.148tu	19.125 ± 0.53wxy	11.825 ± 0.445aa	87.475 ± 0.65ab
2	40.71 ± 0.62mn	35.95 ± 0.127op	30.89 ± 0.735rs	36.175 ± 1.421op	12.235 ± 0.37aa	34.37 ± 1.08opq	47.525 ± 0.714jk	31.93 ± 0.113q	33.015 ± 0.233pqr	14.4 ± 1.555zaa	89.265 ± 0.869a
3	48.285 ± 1.718j	44.935 ± 1.053kl	42.31 ± 0.480lm	40.175 ± 0.007mn	12.675 ± 0.657aa	39.595 ± 0.784mn	49.89 ± 0.268ij	37.52 ± 0.692no	41.485 ± 1.859m	18.115 ± 1.223xy	88.92 ± 0.325a
5	58.575 ± 0.106g	56.115 ± 0.65gh	53.06 ± 0.028hi	53.98 ± 0.084h	17.545 ± 0.671yz	59.255 ± 1.265fg	62.425 ± 0.304f	54.365 ± 1.110h	54.62 ± 0.735h	21.535 ± 0.728vw	89.34 ± 0.721a
10	83.48 ± 1.654c	84.54 ± 0.66bc	76.525 ± 0.714d	72.16 ± 0.028e	30.03 ± 0.452rs	79.53 ± 0.692d	88.475 ± 0.304a	76.92 ± 0.183d	71.8 ± 0.537e	33.31 ± 0.707pqr	88.37 ± 0.028a

Different letters in the column indicate significant differences (*P* < 0.05). EM, ethanol-maceration; MM, methanol-maceration; E_50_M, ethanol:water (50:50)-maceration; M_50_M, methanol:water (50:50)-maceration; WM, water-maceration; EU, ethanol-ultrasonic; MU, methanol-ultrasonic; E_50_U, ethanol:water (50:50)-ultrasonic; M_50_U, methanol:water (50:50)-ultrasonic; WU, water-ultrasonic.

### *β*-Carotene bleaching system

**Figure 2:**
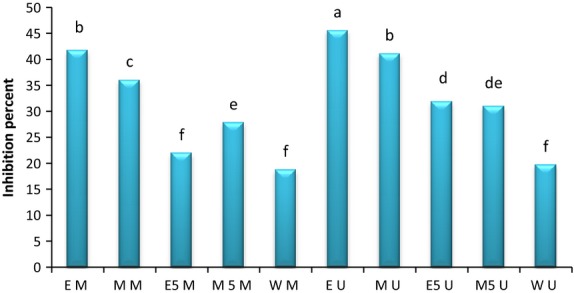
Inhibition percent in *β*-caroten-linoleic acid system. EM, ethanol-maceration; MM, methanol-maceration; E_50_M, ethanol:water (50:50)-maceration; M_50_M, methanol:water (50:50)-maceration; WM, water-maceration; EU, ethanol-ultrasonic; MU, methanol-ultrasonic; E_50_U, ethanol:water (50:50)-ultrasonic; M_50_U, methanol:water (50:50)-ultrasonic; WU, water-ultrasonic.

Synthetic radicals like DPPH are important tools to show the antioxidant activity power, however, they do not use biological oxidizable substrate, so no direct information is determined from extract inhibition activity (Dorman et al. 2003). That is why we measured antioxidant activity of extract in the water:oil *β*-carotene/linoleic acid emulsion. In this method, peroxyl free radicals are made from linoleic acid oxidation due to the absorption of hydrogen atoms from diallylic methylen groups of linoleic acid. These radicals will oxidize unsaturated *β*-carotene. *β*-carotene oxidation minimized when there was an encounter with antioxidants. The antioxidants of extract will decompose produced hydroperoxide in the system. Therefore, the amount of *β*-carotene decomposed is related to the antioxidant activity of extract (Mohdaly et al. 2011). The effect of sesame cake extracts on the *β*-carotene oxidation has been shown in Figure 2. It is obvious that the presence of antioxidant in the sesame cake decreases *β*-carotene oxidation. The extract could be able to scavenge free radicals in the heterogenous medium. So these extracts may be used as antioxidant retentive in emulsion systems (Osawa 1994). The extracts obtained from different solvents, show different degrees of antioxidant activity. EU with the inhibition percent of 45.64 showed maximum antioxidant activity with significant differences than others. EM and MU with no significant differences were after that. The lowest amount of antioxidative activity with no significant differences belonged to WM and WU with the amount of 19.1 and 19.78. Jayaprakasha et al. (2001) mentioned that ethanolic extract of Camelina showed higher antioxidative activity than methanolic one in the *β*-carotene/linoleic acid system, although in DPPH assay methanolic extract was more effective. They mentioned that ethanolic extract contained less polar antioxidants, in addition, butylated hydroxy toluene as a nonpolar synthetic antioxidant indicated higher inhibition. These results together suggest that the polar antioxidant existent in the aqueous phase of emulsion were in the lipid phase with lower concentration. So they were less effective to protect emulsified linoleic acid. While lipophilic antioxidants due to their higher concentration in a lipid phase, indicated higher activity in the emulsion (Moure et al. 2001; Terpinc et al. 2011). These antioxidants concentrate in a oil:air surface and ensure higher protective effect on emulsion (Koleva et al. 2002). As a consequence, this assay just indicates the effect of lipophilic antioxidant, because the medium of this method acts as oil in water emulsion system (Miraliakbari and Shahidi 2008). Suja et al. (2005) measured antioxidative activity of sesame cake methanolic extract by *β*-carotene/linoleic acid assay. They mentioned that the inhibition percent in a concentration of 100 and 200 ppm of extract were 41.7 and 46.6.

### Ferric reducing/antioxidant power

**Figure 3:**
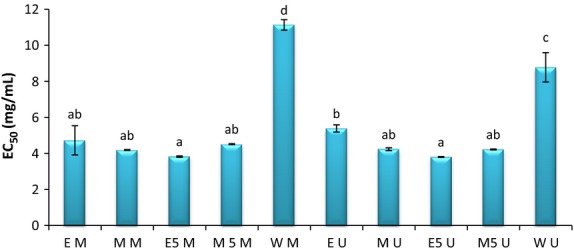
EC_50_ (mg/mL): effective concentration at which the absorbance is 0.5. EM, ethanol-maceration; MM, methanol-maceration; E_50_M, ethanol:water (50:50)-maceration; M_50_M, methanol:water (50:50)-maceration; WM, water-maceration; EU, ethanol-ultrasonic; MU, methanol-ultrasonic; E_50_U, ethanol:water (50:50)-ultrasonic; M_50_U, methanol:water (50:50)-ultrasonic; WU, water-ultrasonic.

Reducing power is often used as an indicator of electron donation which is the important mechanism to determine antioxidative activity of phenolic compound. Presence of reductants like antioxidants in tested sample will reduce Fe^3+^, so reducing capacity of antioxidant is an indicator of its antioxidative activity. The yellow color of the sample due to the extract concentration is changed to different halo of green and blue (Terpinc et al. 2012). The mechanism of reduction is breaking down the free radical chain and donating hydrogen atoms which depends on the antioxidative activity of reductant (Jamuna et al. 2010). FRAP assay is usually used for measuring antioxidative capacity of hydrophilic compounds (Pérez-Jiménez et al. 2008). So, in this research hydro-alchoholic and aqueous extracts in this assay indicated more antioxidative activity than other methods. Antioxidative activity was also increased with increase in extract concentration as can be seen in Table 5, after BHA as a synthetic antioxidant, ethanol:water (50:50) extracts in both maceration and ultrasonic extraction with no significant differences show the highest absorption in 700 nm with the amount of 1.132 nm ± 0.031 and 1.0745 nm ± 0.002 in 10 mg/mL, so the highest antioxidative activity related to ethanol/water (50:50) extracts. M_50_U with the amount of 0.978 ± 0.007 in 10 mg/mL was in the next degree. M_50_M, MU, and MM with no significant meaning were after that. These findings are in agreement with Trabelsi et al. (2010), the ethanol/water (50:50) extract of *Limoniastrum monopetalum* leaves was more effective than methanol/water (50:50) extract due to polarity and the nature of antioxidant. Despite the hydrophilic nature of this assay, aqueous extract shows the lowest antioxidative activity among extracts, but in comparison with other methods, antioxidative activity of aqueous extracts was higher. Generally, the lowest significant differences seen among extracts in this method are in agreement with Pe′rez-Jime′nez and Fulgencio (2006). They mentioned that FRAP has the lowest sensitivity to the solvent type and in proportion to other assays like DPPH, lower differences were seen among extracts in this assay. In this method, EC_50_ is the concentration of extract in which extract absorption reached 0.5. E_50_U and E_50_M in the concentration of 3.805 ± 0.21 mg/mL and 3.827 ± 0.038 mg/mL reached the absorption of 0.5. So, they show the highest antioxidative activity. As can be seen in Figure 3, WU and WM had the lowest antioxidant activity with the highest amount of EC_50_.

**Table 5:** Reducing power of extracts

Samples Conc mg/mL	Extract absorption in 700 nm										

	EM	MM	E_50_M	M_50_M	WM	EU	MU	E_50_U	M_50_U	WU	BHA
1	0.2125 ± 0.003	0.215 ± 0.0063	0.16 ± 0.0012	0.185 ± 0.0063	0.0955 ± 0.0063	0.187 ± 0.0106	0.196 ± 0.0056	0.191 ± 0.0014	0.189 ± 0.0070	0.097 ± 0.0155	2.652 ± 0.0671
2	0.255 ± 0.007	0.3505 ± 0.0077	0.313 ± 0.0056	0.315 ± 0.0063	0.1175 ± 0.0091	0.285 ± 0.0077	0.309 ± 0.0077	0.348 ± 0.0084	0.313 ± 0.0091	0.1145 ± 0.0120	2.694 ± 0.081
3	0.496 ± 0.0091	0.427 ± 0.0056	0.469 ± 0.0098	0.385 ± 0.0070	0.136 ± 0.0065	0.414 ± 0.0056	0.46 ± 0.0070	0.424 ± 0.0091	0.418 ± 0.0098	0.1515 ± 0.0077	2.612 ± 0.364
5	0.556 ± 0.0091	0.585 ± 0.0056	0.649 ± 0.0077	0.56 ± 0.0071	0.192 ± 0.0084	0.507 ± 0.0108	0.596 ± 0.0091	0.662 ± 0.095	0.609 ± 0.0077	0.380 ± 0.0063	2.983 ± 0.0042
10	0.667 ± 0.0098	0.931 ± 0.0077	1.132 ± 0.031	0.931 ± 0.0056	0.460 ± 0.0077	0.730 ± 0.0059	0.930 ± 0.013	1.0745 ± 0.0021	0.978 ± 0.0077	0.475 ± 0.0065	3.176 ± 0.103

EM, ethanol-maceration; MM, methanol-maceration; E_50_M, ethanol:water (50:50)-maceration; M_50_M, methanol:water (50:50)-maceration; WM, water-maceration; EU, ethanol-ultrasonic; MU, methanol-ultrasonic; E_50_U, ethanol:water (50:50)-ultrasonic; M_50_U, methanol:water (50:50)-ultrasonic; WU, water-ultrasonic.

## Conclusion

Ultrasound-assisted extraction is one of the new, simple, and inexpensive extraction methods that is used to extract phenolic compounds of sesame cake and compared with maceration method. It was observed that extraction method and solvent type affected phenolic compound extraction. Since ultrasound wave generated cavitation phenomenon, mass transfer increased, and therefore more phenolic compound can be extracted from disrupted tissue. Solvent properties like viscosity, surface tension, and vapor pressure influence cavitation occurrence and hence on phenolic extraction. In this research, three different antioxidant activity methods were done to evaluate antioxidant activity of extracts, since the nature of these methods are different, different responses were observed among extracts, such that in DPPH assay, MU extract, in *β*-carotene bleaching system, ethanol-ultrasound, and in FRAP method ethanol/water (50:50)-ultrasound and ethanol/water (50:50)-maceration were the best extracts. Considering TPC assay and antioxidant capacity, ethanol-ultrasonic and methanol-ultrasonic extracts were considered as the best extracts.
